# Unmet needs in functional neurological disorder related to digital healthcare

**DOI:** 10.1098/rstb.2024.0460

**Published:** 2026-09-17

**Authors:** Jon Stone

**Affiliations:** Centre for Clinical Brain Sciences, University of Edinburgh, Edinburgh, UK

**Keywords:** functional neurological disorder, conversion disorder, digital healthcare, diagnosis, treatment

## Abstract

Functional neurological disorder (FND) is a common yet historically neglected condition that presents major unmet needs across diagnosis, treatment and health service delivery. This article examines how digital healthcare could address gaps in several areas: (i) using large biobank and clinic datasets to reveal epidemiology, comorbidity, mechanisms, economic burden and possibly treatment effects; (ii) augmenting complex and time-consuming assessment processes that currently exceed clinical capacity with artificial intelligence; (iii) using digital tools to improve diagnostic precision—for example through automated tremor analysis, quantification of functional motor signs and speech recognition; (iv) improving self-management and access to therapy via online tools and telehealth programmes; (v) improving outcome measurement and existing therapy using wearables and telehealth; and (vi) new therapies such as AI-assisted therapies, biofeedback and virtual or augmented reality interventions. Finally, I discuss the risks of depersonalization, privacy breaches, therapeutic substitution, digital dependence and digital exclusion. In addition, positive therapist effects—variability in outcomes driven by the clinician rather than the intervention—remain difficult to replicate digitally. The challenge for clinicians and researchers is to ensure that technological advances humanize rather than mechanize care, harnessing digital tools to extend expertise, consistency and accessibility without eroding the therapeutic relationship at the heart of effective FND treatment.

This article is part of the discussion meeting issue ‘Digital healthcare for the management of functional neurological disorders’.

## 1. Introduction

Functional neurological disorder (FND) is one of the commonest conditions in neurological practice, affecting around 50–100 000 people in the UK [1]. It has been historically neglected, and as a consequence, it is associated with a large number of clinical and research unmet needs, which digital healthcare could potentially meet. In this article, I outline some key areas of unmet need and review where digital technologies have already shown some promise or may do so in the future.

I have organized this article using headings that represent some areas of unmet need in epidemiology, assessment, diagnosis, triage, access to treatment, outcome measurement and delivering existing therapy and new therapy. Finally, I will discuss some of the potential downsides of using digital technology in the management of FND. My sincere hope is that the pace of current technological change will make this article, or at least parts of it, look redundant in a few years.

## 2. Making the case for services through large datasets

### (a) The unmet need

Clinicians and patients still face challenges of convincing those organizing healthcare that FND is as common as the data suggests. Services for people with FND around the world are very patchy and often non-existent. In many places in the world, people with FND are specifically excluded from rehabilitation services, and typically, many neurological centres do not regard it as a core condition that they should be responsible for [2].

As an indication of its low profile, I began the first general neurology subspecialist FND clinic in the UK in 2005 (acknowledging a specialist functional seizure clinic in the UK before that started by Rod Duncan in Glasgow). When I started a self-help website for people with FND in 2009, neurosymptoms.org, there was no online community of people with FND and no patient-led organizations anywhere globally. FND had virtually no visibility in neurology textbooks or curricula [3] and, under the name conversion disorder, was typically a brief entry in psychiatric and other textbooks. News stories about FND appeared, but typically with the patient clearly misdiagnosed with another neurological condition, such as epilepsy [4]. We are still waiting for a global celebrity to declare that they have FND or campaign on behalf of it, even though statistically, many celebrities must have been diagnosed with the condition. Coding of FND has been historically poor and remains so. In one study, US pediatric neurologists only coded for FND in 23% of occasions when they had made this diagnosis [5]. In many health care systems that rely on payment by coding, the fact that codes for FND have always been in mental health/psychiatry categories has meant that clinicians working in neurology have often been reluctant to accurately code FND. For example, neurologists known to me in a wide variety of countries, such as the USA, Russia and Australia, have been in the routine habit of coding patients they know have FND as ‘Leg weakness - cause unknown’ or ‘Encephalopathy’ to enable payment under neurological coding. There have also been concerns about the consequences of misdiagnosing someone with a stigmatized condition [6].

It is not surprising, given all these issues, which are only now beginning to change, that FND suffers from a lack of awareness and prioritization in healthcare systems. A major unmet need is robust, large-scale data showing the frequency of FND and its consequences on the population's quality of life and the global economy.

### (b) Solutions from digital healthcare

One key argument for better services is to have better data. Data on frequency, disability, cost and the cost of not treating through payments in disability and unemployment benefits.

FND is on the threshold of benefiting from big data. We are just starting to see data emerging from the large TriNetX clinical database relating to FND. The largest cohort study to date in FND was a meta-analysis of studies globally on functional movement disorder, totalling 4 905 cases [7]. The first FND study from the TriNetX research network reports on 220 312 individuals with functional neurological disorder, giving an estimate of the risk of autism (six times the population), which is not only a really important clinical question, but one which would simply be impossible to gain through standard epidemiological research [8]. Large economic datasets are starting to make the case for the costs of FND, too. A US database has shown levels of spending on FND over $2 billion, with a 47% increase over 2 years, probably related to increased diagnosis [9].

The possibilities for more accurate epidemiology that can, in turn, also help us understand the aetiology and natural history of this condition are extraordinary. Very large datasets could also disentangle treatment effects as well as the impact of socio-economic factors and provide real-world costs. Links with genomic or imaging repositories are already creating an enormous impact in understanding the basis of conditions like chronic nociplastic pain. For example, a recent analysis from the UK Biobank showed in 523 000 people that genetic and imaging biomarkers predicted pain from structural disorders but not those associated with self-reported pain (nociplastic pain), but that these same biomarkers worked in synergy with psychosocial factors to predict self-reported pain [10]. Databases could also potentially help overcome the difficulties and expense of randomized controlled trials of rehabilitation. Although they are the ‘gold standard’, randomized trials in rehabilitation suffer from several issues, especially the problem of how to select a control group, the impossibility of blinding and short durations of follow-up. Large datasets and methodologies like target trial emulation, or precision medicine treatment selection, are promising methodologies that may help provide other forms of evidence of benefit [11,12].

Large datasets are not a panacea. Along with well-known issues of granularity, representativeness and coding, there are particular problems with the accuracy of phenotyping. This is something that is especially problematic for FND, where we have diagnostic labels like ‘seizure’ which often hide both epilepsy and functional seizures, or ‘cognitive decline’ which hides both early dementia and functional cognitive symptoms.

## 3. Overcoming constraints of time and complexity in clinical assessment using artificial intelligence

### (a) The unmet need

Individuals with FND are typically multimorbid with neurodevelopmental, other neurological, medical and psychological comorbidities that interact with a set of predisposing, precipitating and perpetuating factors that are unique for each individual. An ideal assessment of someone with FND includes all current physical and mental health symptoms, a description of daily social and occupational activity, a detailed life narrative which encompasses developmental history, school, family life, occupational history and personal medical history, complemented by records of medical history from birth (and especially since teenage years). Ideally, the assessor needs to understand how various physical or psychological events may have been relevant to the onset or maintenance of FND symptoms, and also how other functional disorders and comorbidities have interacted over time to lead to the current situation. An assessment should also encompass a discussion with the patient and their friends and family about their ideas and expectations about their condition and its treatment. Finally, a physical examination needs to be integrated with existing and planned investigations to come to a diagnosis, typically involving overlapping diagnoses of different functional symptoms and disorders and comorbidities. Later, that diagnosis needs to be expanded into a therapy-relevant formulation, preferably done cooperatively with the patient and which serves as the entry to treatment.

This process is inescapably time-consuming. The reader may feel exhausted just reading that description of the assessment. My own experience is of seeing people with FND in time scenarios ranging from 10 to 20 minutes in a time-pressured emergency setting, up to 5 hours for a medicolegal assessment when every element of the list above can be explored in detail. A research question to be answered is how much benefit and harm are avoided, with a longer versus a shorter assessment. My anecdotal experience is that the longer you have, the more likely you are, on average, to alter the outcome. Surgeons have long been able to assert their need to take the time required to carry out their treatment safely. If it takes 7 hours to take out someone's pituitary gland, that's generally how long they take to do it.

Unfortunately, such long assessments are beyond the reach of most health services when it comes to FND but well within the scope of AI. If AI has taken the history, does that mean that the explanation and formulation can be made as well by a human who has not sat through it? Does the patient even need a human to do that, especially if it does it with more patience, thoroughness and accuracy than a human? What parts of the examination still need a human to do them? These are all research questions, where we will start to see answers to more common mental and physical health conditions before we get to FND.

### (b) Solutions from digital healthcare

AI promises a naturalistic and conversational way to acquire data from people with complex health conditions. Many people are already using software like ChatGPT as a substitute or adjunct for assessment and therapy. At a basic level, AI may help speed up the process of summarizing consultations for clinicians and provide better access to patients about what was said [13], although there are plenty of warnings about how the reality could be bloated and of incomprehensible records, which mislead or fail to do justice to a person's story [14]. AI software can be readily adapted for specific purposes and trained on specific assessment or therapy manuals, for example, meaning there will be high fidelity to treatment. AI therapy has no time limits and does not need an appointment. Many services, such as ChatGPT and Gemini, are currently free. An AI clinician offers an endlessly patient, objective and remarkably accurate diagnostic viewpoint [11,15], and work is moving at pace for more specific AI trained on medical consultation skills [16]. For people with FND, many of whom have anxious avoidance such as agoraphobia or neurodiversity, therapy from a phone may seem more accessible than the conventional variety.

On the other hand, it remains to be seen to what extent a face-to-face human connection is essential for assessment and treatment. Therapists have also noted that AI clinicians may be ‘too perfect’, and that their current tendency to a reassuring and accepting tone may not be therapeutic, or even be harmful, for example, fostering dependency, amplifying false or delusional ideas, or failing to adequately challenge someone with health anxiety or obsessive compulsive disorder (OCD) [17]. If an agent does not give a desirable answer, the user can alter the prompt and try again. Can AI really substitute for core aspects of the consultation? If somebody has a need to ‘be heard’ by a health professional and ‘taken seriously’, will it really count for them if these needs are met by AI, however elegantly? Although many AIs are free, reliable clinical AIs will inevitably be under market forces, which could ultimately accentuate health disparities.

Personally, I had initially been sceptical that I could be replaced by a digital agent, but increasingly it is becoming clear how immensely powerful these tools are. Where most people are landing at present is for AI to be complementary, integrating into current clinical practice and helping where it can, but not removing the human component of treatment [11].

## 4. Improving and standardizing diagnostic precision in functional neurological disorder

### (a) The unmet need

FND is a clinical diagnosis, ideally made by a health professional with expertise in neurological conditions. Like other clinical diagnoses, such as Parkinson's disease, migraine or depression, it relies on clinical judgement. It especially depends on pattern recognition, where characteristic features are grouped and interpreted in a Bayesian way to reach a confident diagnosis. For functional seizures, for example, clinicians weigh seizure semiological features, witness history and subjective experiences against pre-test probabilities. For movement disorders, diagnosis depends on direct examination, including manual testing of power, stance and gait, and observing interactions such as Hoover's sign or the tremor entrainment test.

Follow-up studies suggest that whatever neurologists are doing, the diagnosis of FND appears reasonably stable over time [18,19]. Yet the field of FND suffers at present from a lack of precision and standardization in how we describe many of the clinical features that we say are typical of our diagnoses, and from the absence, for many symptoms, of objective tools to quantify or test their diagnostic performance. When I see colleagues testing for tremor entrainment or carrying out a Hoover's sign, I notice differences in the way they do it compared to me. In short, the diagnosis of FND depends heavily on an often-undefined neurological ‘expertise’, which can differ substantially between examiners.

This problem becomes especially evident when considering the question of FND comorbidity. How can we meaningfully study the comorbidity of FND in people with multiple sclerosis, Parkinson's disease or spinal disorders unless we define FND clinical signs more objectively? Every FND sign has limits in terms of sensitivity and specificity. Hoover's sign, for example, is evidence of impaired agency, of which FND is the commonest cause, but is also positive in individuals with apraxia or neglect [20,21].

Clinically, this may not pose a huge problem if we want our diagnoses to be as accurate as other conditions in neurological practice. After all, while several scales exist for diagnosing Parkinson's disease, most clinicians rely on experienced clinical assessment rather than formal scoring systems owing to time constraints.

The unmet need, therefore—particularly for research, and especially for studies of comorbidity—is for technology that can standardize and objectify the assessment of FND signs. Such tools should minimize dependence on individual neurological expertise, offer reproducible and quantitative measures, and be capable of detecting a range of clinical features to support reliable diagnosis.

The research community should also be open-minded to the possibility that other indicators from the history [22] or biomarkers [23] may also provide an accurate diagnosis when filtered through machine learning.

### (b) Solutions from digital healthcare

There are already some examples of digital technology meeting the unmet need of standardization in FND diagnosis. Petra Schwingenschuh *et al.* [24] validated an electrophysiological test battery in a large sample of 38 individuals with functional tremor and 73 individuals with other forms of tremor, including essential tremor, Parkinson's disease and dystonic tremor. They showed that a 10-point score based on tapping performance, entrainment, pause with ballistic movements, tonic co-activation, coherence of bilateral tremor and ‘total power’ (a surrogate of tremor amplitude) had a sensitivity of 90% and a specificity of 96%. They also considered that the battery was useful for hard-to-diagnose patients. It is interesting to reflect that despite its comprehensive nature and publication in 2016, this battery has not been repeated in subsequent studies. They comment that it only needs two accelerometers, EMG recording facilities, a metronome, a 500g weight and 15–30 minutes per patient. In reality, this is an investment in time, skill and equipment unavailable to most clinicians or even researchers to confirm a diagnosis that is often readily apparent clinically. It indicates the need for digital solutions that are much quicker and more available.

Many authors have investigated the use of ‘computer vision’ to diagnose tremor [25]. The application of functional tremor using computer vision on a smartphone has only been described, however, in a single patient in 2019 and 2021 [26].

Neurophysiological approaches to the measurement of Hoover's sign, one of the most reliable and commonly used clinical signs of FND, have been recorded in several small studies, using a weigh scale and EMG [27,28]. Our own group has developed an adaptation of a digital weigh scale and a graphic user interface and has started testing Hoover's sign in healthy controls in preparation for clinical testing, with a view to researching the utility of the sign as a feature of FND comorbidity.

Functional speech disorder lends itself strongly to automated detection. A review of voice recognition in neurological disorders showed that the technique has been used in a wide range of neurological and psychiatric disorders, including depression, schizophrenia, multiple sclerosis and Parkinson's disease [29,30]. It seems probable that functional speech disorder would lend itself well to automated detection, especially to look for the internal variability commonly seen in functional disorders of phonation, fluency and articulation that is the hallmark of the condition. When I worked with Nick Miller on recordings of people with foreign accent syndrome a few years ago, I was astonished at the typical FND clinical features in terms of language, voice quality, prosody and fluency that he was able to pick out from the recordings using his years of experience [31,32].

Functional seizures also have many features that are susceptible to automated diagnosis, both in the witness history, subjective symptoms, objective semiology and EEG findings. For example, a study of 300 patients with epilepsy, functional seizures and syncope found that 36 questions determined by machine learning could distinguish syncope from the other two with 100% sensitivity, although only 86% of patients with epilepsy and 75% of those with functional seizures were correctly identified [33]. Such data may provide diagnostic aid but are too unreliable for a non-expert diagnosis. A neural network trained on EEG data managed an accuracy of 87% on 107 individuals being investigated for epilepsy or functional seizures [34]. Automated analysis of formulation effort using transcripts of recordings only managed successful classification in 71%. This figure is lower than a range of studies which have used data such as gender, comorbidities and seizure features such as eye closure, which produce areas under the curve of around 80–85% [35,36]. The functional seizure literature, therefore, indicates that objective classification using technology is possible, but has yet to reach levels that do more than assist in diagnosis. Direct computer vision of episodes, mimicking smartphone diagnosis, now increasingly relied upon by clinicians [37–40], must surely be coming soon as a low-cost and affordable alternative that requires further investigation.

**Figure 1.**
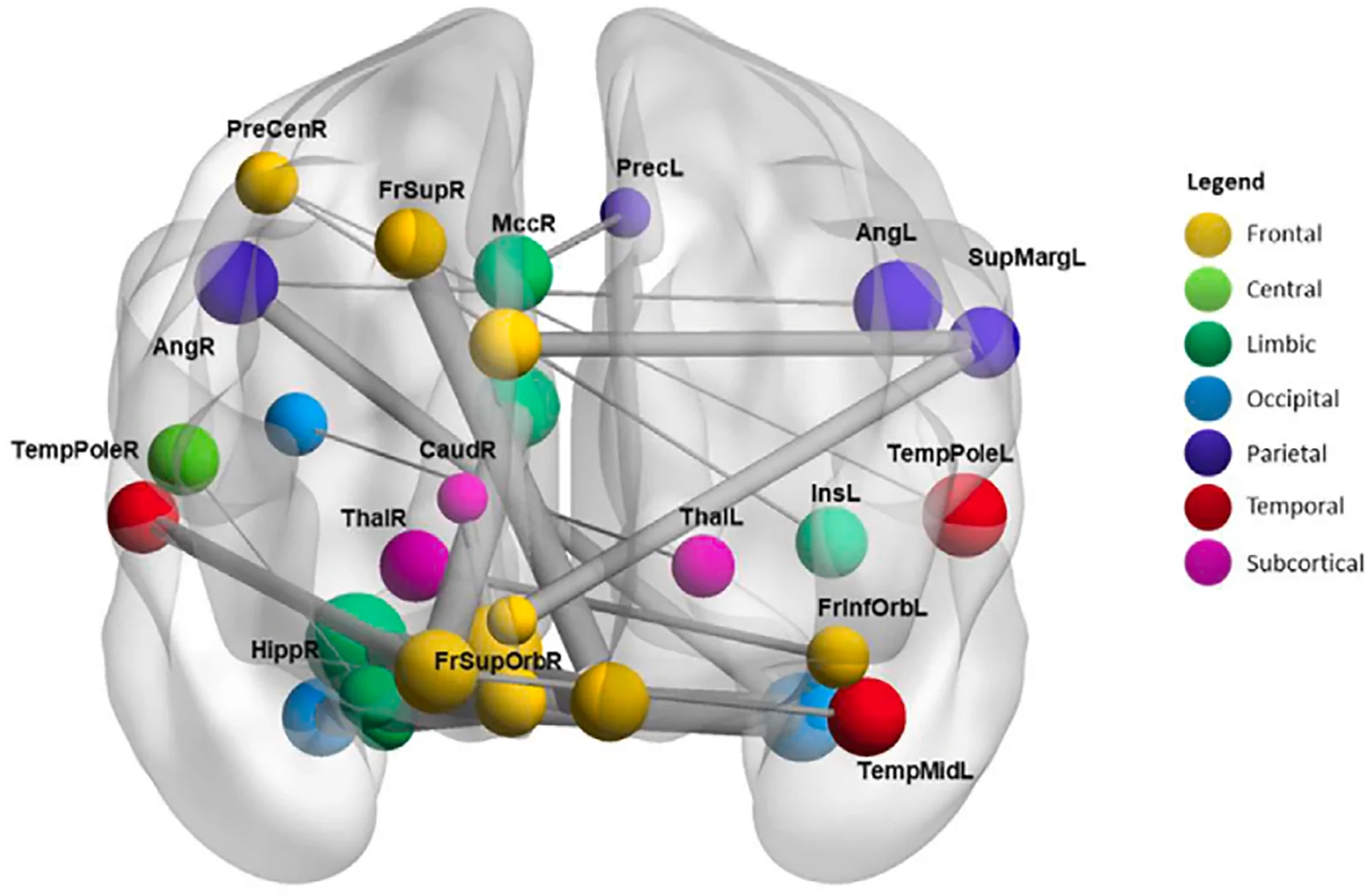
Regions yielding the most discriminative connections in a study of 86 patients with FND and 87 healthy controls, having whole-brain resting-state functional MRI. The groups were distinguished with imaging alone, with a pooled accuracy of 72%. Reproduced with permission from Weber *et al.* [41].

Finally, might it be possible to diagnose FND just by analysing an fMRI scan? A study of 86 FND patients and 87 healthy controls using whole-brain resting-state functional connectivity was able to distinguish the groups with a pooled accuracy of 72% [41] (figure 1). Again, a long way from a diagnostic test, and a finding that could easily arise as a result of a related comorbidity rather than specific FND symptoms, but an interesting gauntlet to the research community. This is discussed further by Selma Aybek and colleagues elsewhere in this issue [42].

## 5. Improving self-help management, formulation, triage and access to treatment

### (a) The unmet need

For an experienced clinician, the diagnosis of FND is in some ways the easy bit. I have described the time-consuming nature of assessment, but the next phase is explanation, formulation and triage. After triage, the patient may well wait for some time for therapy. What can they do to help themselves if they are waiting for in-person treatment?

Why has this particular person got FND? Is now the right time for treatment, and what treatment is most likely to help? Can we tell the patient what their chances are of improving?

This part of the clinical management of the patient with FND may be started by the diagnosing clinician or perhaps by a psychiatrist, psychologist or other therapist to whom they have been referred.

Ideally, the formulation of why FND symptoms have occurred is jointly produced by the patient and clinician. They then jointly decide if they would like to proceed to therapy and what kind of educational material they would like to have as part of that process, for example, video, books or reading webpages. They may specifically want or not want to engage with AI.

In the real world, there is often insufficient time for these important conversations. Referrals are often made in a ‘knee-jerk’ way, and the patient is left bewildered by their diagnosis, its treatment and with little idea of how therapy may help. In some cases, referral to treatment occurs in a situation where the patient remains deeply unsure that the diagnosis is even correct, or at a time in their life when it is very hard for them to manage the commitment to treatment.

### (b) Solutions from digital healthcare

A trial of internet-based self-help therapy for 186 people with functional motor disorder in the Netherlands showed that people valued having relevant and detailed information [43]. But this information made no difference to self-rated health at 3 or 6 months. The important lesson here is that information alone is not enough, even though it is an essential platform for treatment.

Another feasibility study of an online educational intervention for 30 individuals with functional cognitive disorder, positioned not as therapy, but containing many self-help elements, showed high levels of satisfaction and some promising signals on illness perceptions and quality of life, while five individuals were dissatisfied with the lack of personalization and human contact [44].

**Figure 2.**
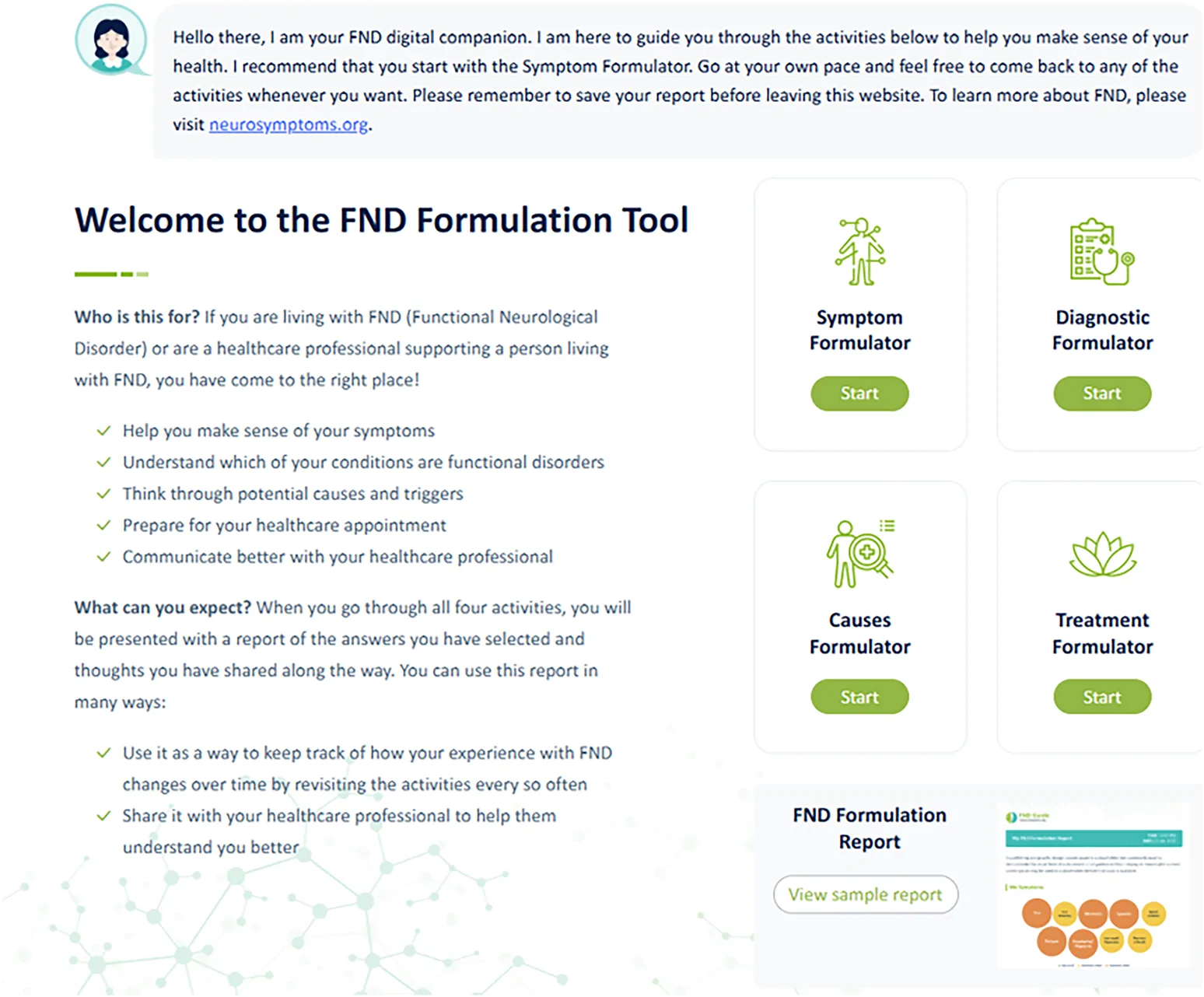
FND formulator tool on neurosymptoms.org as an example of digital co-formulation.

In my own practice, I have made a digital tool called the FND formulator, available freely at www.neurosymptoms.org, which aims to improve at least one part of this process (figure 2). By asking the patients themselves to complete a series of questions about their symptoms, their views on the reasons why they may have developed FND and their views on the diagnosis and treatment, the clinician may gain more granular knowledge of the patient's current views. A formulation that is co-constructed using tools like this is likely to be one that a patient is more likely to sign up to. It also allows formulation to be revised over time, as the process of therapy can often alter an individual's views about their own vulnerabilities. Typically, I use this along with signposting to reading material about the patient's individual symptoms using a ‘recommender’ function on the website.

It is possible to envisage a much more sophisticated AI-based approach to this stage of FND treatment, where an individual would receive a digital education programme tailored to their own symptoms, and produce a formulation, perhaps at the end of that process, once they have learnt more about the condition and the forms of treatment available. Such a process would enable clinicians to have a much more nuanced view of the unique circumstances of their patients. In particular, it seems essential to establish an individual's motivation for further therapy and not assume that it is necessarily the right time for them to engage in treatment. Therapy is an expensive resource, and local experience suggests that many referrals for FND-related therapy are premature or inappropriate when not carried out by experienced clinicians.

## 6. Improving outcome measurement

### (a) The unmet need

Measuring outcome in FND is problematic. A condition with numerous possible core and associated symptoms and a massive spectrum of disability and distress. A condition in which subjective distress and disability from symptoms like fatigue, pain and cognitive symptoms can sometimes be at odds with external normality, or conversely, where external abnormality, such as seizures, gait or speech issues, can be at odds with internal states of improved health.

The theoretical complexities [45] and existing literature on outcome measures [46] have led to consensus recommendations which suggest the prioritization of subjective outcome measures over objective ones, but also call for the development of new measures that may be more FND-specific.

A further problem in outcome measurement lies in the episodic nature of many symptoms. A single clinic snapshot can miss the day-to-day fluctuations that determine disability and quality of life. Moreover, outcome measurement is typically labour-intensive, dependent on clinic attendance and prone to recall bias.

### (b) Solutions from digital healthcare

Digital healthcare offers ways to capture both subjective and objective change at a scale and resolution not previously possible. Ecological momentary assessment involves ‘in-the-moment’ questions to help monitor symptoms in real time and in relation to context. Wearables, smartwatches and phones can measure many variables, including FND-specific ones such as tremor, speech or typing. There are clear advantages to such approaches in terms of giving patients and clinicians accurate data and perhaps detecting trends that might indicate imminent relapse or unrecognized stress. There are also disadvantages. Does it matter if the wearables indicate improvement if the person feels worse? Could endlessly monitoring symptoms or answering questions about health actually worsen people with FND who already have excessive bodily hypervigilance? This topic is discussed in more detail by Susannah Pick and Matthew Hotopf in another article in this issue [47].

## 7. Improving existing and new therapies

### (a) The unmet need

Access to therapy is currently highly variable for people with FND. For example, a survey of 126 commissioning groups and health boards in the UK in 2022 found that 50% had no specific agreement to treat FND or did not treat FND. Between 14 and 43% of services did not accept physiotherapy, occupational or psychology referrals for people with FND [2]. Are there ways in which digital technology could improve access to therapy regardless of postcode? And finally, in what ways can existing therapies be enhanced by digital technology?

Our existing therapies for FND have created optimism among clinicians and patient-led organizations [48,49]. But the reality is that we need more effective treatment. Many patients only experience improvement rather than resolution of their symptoms, and many are not able to benefit. New forms of therapy which are specific to FND symptoms, and which are scalable and accessible, are urgently needed.

### (b) Solutions from digital healthcare

Virtual and telehealth therapy for FND has already been trialled in a number of locations and appears to be helpful [50]. For example, 32 US veterans with functional seizures showed seizure reductions and improvement in quality of life and health status using a remote-only platform [51]. Retraining and Control Therapy (ReACT), a variation on third-wave cognitive behavioural therapy for FND using principles of habit reversal, was successfully applied remotely to 34 widely geographically distributed adolescents in the US after one in-person session, with over 60% reporting seizure or motor symptom freedom at six months [52]. An Italian study of 64 individuals with functional motor disorder shows that a 12-session weekly telemedicine programme following 5 days of inpatient rehabilitation produced better outcomes than a self-management programme alone [53]. An online five-session group for 44 individuals with functional cognitive disorder obtained good attendance rates (81% at least four sessions) and high satisfaction rates, with modest benefits [54].

**Figure 3.**
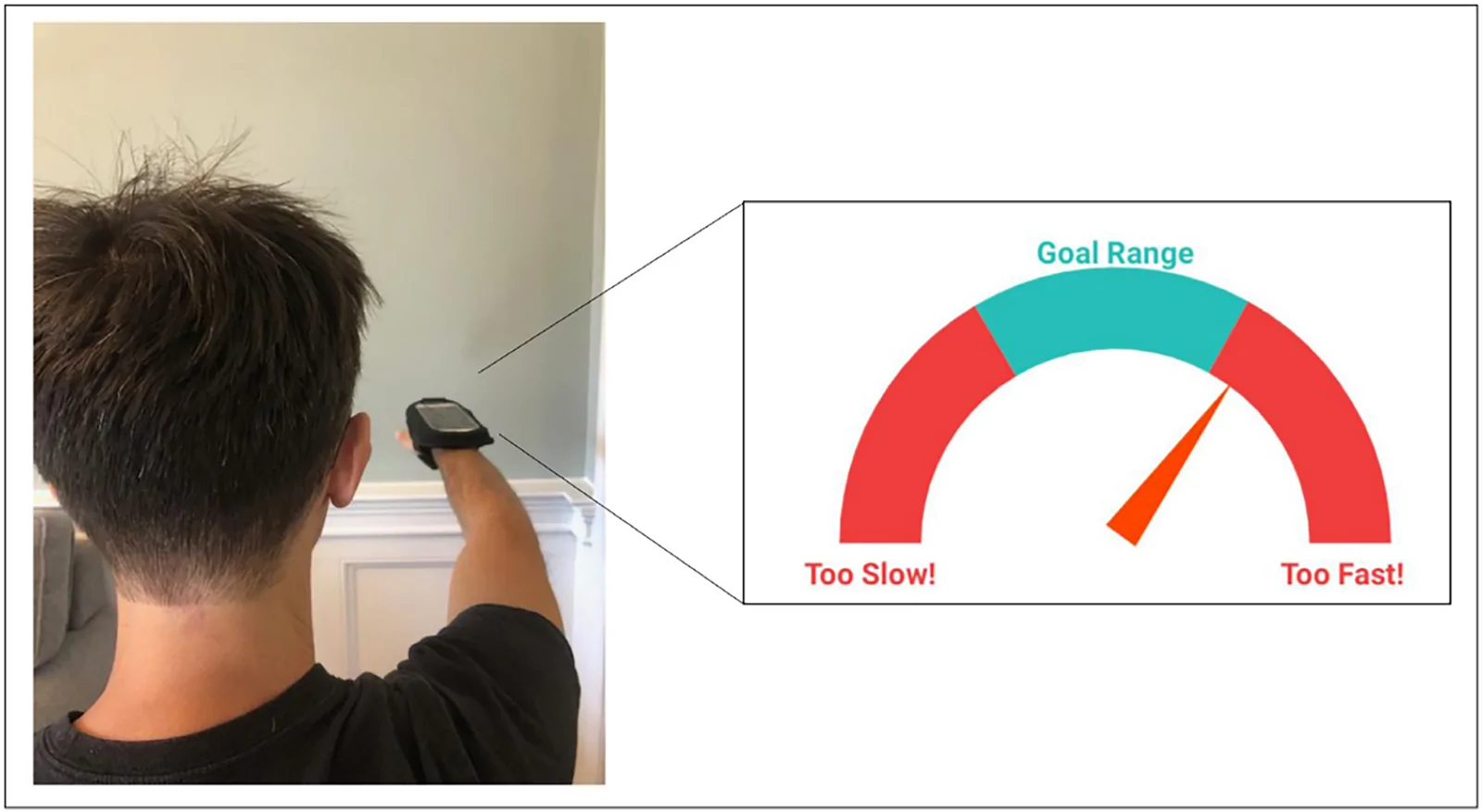
An example of smartphone feedback for functional tremor entrainment. Reproduced by permission from Garris *et al.* [60].
— *Neurofeedback and brain-computer interfaces.* Neurofeedback typically uses components of an EEG signal to provide a visual, auditory or tactile stimulus to an individual, which represents that signal and can be modulated with therapist-provided tasks. Reports of neurofeedback in cases and small case series have been in the literature for nearly 30 years [61]. Neurofeedback is discussed elsewhere in this issue [62].

Several potential new digitally-based therapies are already being explored in early forms by researchers, including:

— *Virtual Reality and Augmented Reality therapy.* Researchers are beginning to explore the possibilities that this technology could offer to FND [55–58]. The promise of altered perceptual experiences offered by these technologies offers obvious promise to powerfully alter internal representations of body ownership and agency, but also risks in terms of potentially exacerbating states of bodily compartmentalization.
— *Digital psychotherapy and conversational AI*. This promises the ability to match individual characteristics of the patients to the therapy. For example, someone with avoidance might begin with digital exposure, whereas someone with trauma or generalized anxiety may need to tackle general arousal initially. In FND, there have been some early descriptions of how a ‘chatbot’ for functional movement disorder might look [59] as well as a description of a semi-individualized online app-based self-help intervention for functional cognitive disorder [44]. I have discussed some of the advantages and drawbacks of this approach in assessment earlier.
— *Biofeedback.* Digitally enhanced biofeedback offers the opportunity to use signals collected by wearables or smartphones to provide real-time feedback to enhance therapy. An example of this is a ‘tremor retrainer smartphone application’ that was trialled on three paediatric patients with functional tremor. It used auditory cues and a visual gauge to provide an entrainment intervention (figure 3) [60].

## 8. The downsides of digital healthcare in FND

For balance, it is worth considering a range of downsides involved in digital healthcare.

— *Loss of human connection.* The placebo effect in clinical trials is evidence of the non-specific benefits of being involved in healthcare, especially when there is a human connection [63]. The therapist effect in psychotherapy trials, which accounts for anything up to 29% of the treatment effect, is evidence that some therapists have a more powerful therapeutic personality than others [64].
— *Digital dependency.* Conversely, some therapists are beginning to find that digital agents can promote dependency, especially if that agent is always accepting and always available [17].
— *Risk of therapeutic substitution.* Digital solutions for therapy are already appearing as substitutes for referral. In my own practice, I see many people who lack enthusiasm for these substitutes. I am struck by how often patients I meet who have had telephone consultations with therapists do not even regard these as appointments, which they describe as ‘just a phone call’. Economic pressures to provide low-cost scalable interventions should not blind us to their real-world appraisal.
— *Privacy and data governance issues.* These will remain an issue in this area, with a risk of catastrophic data leakage in some cases.
— *Digital exclusion.* Especially those who are older, economically disadvantaged, with a different first language or who may have symptoms such as poor concentration or light sensitivity that make it difficult for them to engage with screen-based technology.

## 9. Conclusion

Digital technologies offer huge potential to enhance clinical research, assessment, diagnosis and treatment of FND. The challenge for clinicians is to ensure that use of such tools serves to further humanise, and not mechanise, the care of FND.

## Data Availability

This article has no additional data.

## Theme

One contribution of 12 to a discussion meeting issue ‘Digital healthcare for the management of functional neurological disorders’.
